# The Fat Kidney

**DOI:** 10.1007/s13679-023-00500-9

**Published:** 2023-03-18

**Authors:** Ludovica Verde, Stefania Lucà, Simona Cernea, Cem Sulu, Volkan Demirhan Yumuk, Trond Geir Jenssen, Silvia Savastano, Gerardo Sarno, Annamaria Colao, Luigi Barrea, Giovanna Muscogiuri

**Affiliations:** 1 Department of Public Health, University of Naples Federico II, Naples, Italy; 2 Centro Italiano Per La Cura E il Benessere del Paziente con Obesità (C.I.B.O), University of Naples Federico II, Naples, Italy; 3 Unit of Internal Medicine, Department of Medical and Surgical Sciences, University Magna Graecia of Catanzaro, 88100 Catanzaro, Italy; 4 Technology of Târgu Mures/Internal Medicine I, George Emil Palade University of Medicine, Pharmacy, Science, and Technology of Târgu Mureş, Romania; 5 Diabetes, Nutrition and Metabolic Diseases Outpatient Unit, Emergency County Clinical Hospital, Târgu Mureş, Romania; 6 Division of Endocrinology, Metabolism and Diabetes, Cerrahpaşa Medical Faculty, Istanbul University-Cerrahpaşa, Istanbul, Turkey; 7 Department of Transplantation Medicine, Oslo University Hospital-Rikshospitalet, Oslo, Norway; 8 Dipartimento di Medicina Clinica e Chirurgia, Diabetologia e Andrologia, Unità di Endocrinologia, Università degli Studi Di Napoli Federico II, Via Sergio Pansini 5, 80131 Naples, Italy; 9 General Surgery and Kidney Transplantation Unit, d’Aragona University Hospital, San Giovanni di Dio e Ruggid, 84131 Salerno, Italy; 10 Cattedra Unesco Educazione alla salute e allo sviluppo sostenibile, University Federico II, Naples, Italy; 11 Dipartimento di Scienze Umanistiche, Centro Direzionale, Università Telematica Pegaso, Via Porzio, Isola F2, 80143 Naples, Italy

**Keywords:** Chronic kidney disease, Obesity, Hyperfiltration, Diet, Tirzepatide

## Abstract

**Purpose of Review:**

The purpose of this review is to summarize the current evidence on the role of obesity in the development and progression of chronic kidney disease and the current evidence on nutritional, pharmacological, and surgical strategies for the management of individuals with obesity and chronic kidney disease.

**Recent Findings:**

Obesity can hurt the kidney via direct pathways, through the production of pro-inflammatory adipocytokines, and indirectly due to systemic complications of obesity, including type 2 diabetes mellitus and hypertension. In particular, obesity can damage the kidney through alterations in renal hemodynamics resulting in glomerular hyperfiltration, proteinuria and, finally, impairment in glomerular filtratation rate. Several strategies are available for weight loss and maintenance, such as the modification of lifestyle (diet and physical activity), anti-obesity drugs, and surgery therapy, but there are no clinical practice guidelines to manage subjects with obesity and chronic kidney disease.

**Summary:**

Obesity is an independent risk factor for the progression of chronic kidney disease. In subjects with obesity, weight loss can slow down the progression of renal failure with a significant reduction in proteinuria and improvement in glomerular filtratation rate. Specifically, in the management of subjects with obesity and chronic renal disease, it has been shown that bariatric surgery can prevent the decline in renal function, while further clinical studies are needed to evaluate the efficacy and safety on the kidney of weight reducing agents and the very low-calorie ketogenic diet.

## Introduction

Obesity is a multifactorial chronic disease that has been recognized as one of the most serious public health concerns worldwide. Recent data report that there are about 650 million people with obesity worldwide and that 59% of adults and almost 1 in 3 children from the European Region suffer from overweight or obesity [1]. Obesity is defined as a “silent killer” because it plays a central role in the development of cardiovascular diseases and cancer [2]. In addition, obesity has been associated with decreased health-related quality of life and increased all-cause mortality in the general population [2]. It has been suggested that obesity also is an independent risk factor for the progression of chronic kidney disease (CKD) [3]. In fact, the global estimated prevalence of CKD is 11–13%, which increases concomitantly with the growing prevalence of obesity [4]. Furthermore, CKD is a global health issue because it has been associated with an increased risks of cardiovascular morbidity and mortality [4]. CKD is characterized by albuminuria (≥ 30 mg/day) and/or reduced estimated glomerular filtratation rate (eGFR) < 60 ml/min/1.73 m^2^, persistent for at least 3 months [5]. Indeed, higher body mass index (BMI) is associated with development of proteinuria in individuals without kidney disease [6]. In a study carried out by Qin et al. A total of 41,085 subjects with obesity were included to investigate the correlation between obesity and the urinary albumin-creatinine ratio (UACR) [6]. The main finding of this study was that obesity was associated with higher risk of elevated UACR even after adjusting for multiple risk factors [6]. Furthermore, higher BMI is associated with low eGFR [7]_._ Adipose tissue not only has the function of storing and providing energy in fasting state, but it is also an endocrine organ that can produce adipocytokines, which influence systemic homeostasis [8]. In obesity, excessive production of pro-inflammatory adipocytokines causes chronic low-grade systemic inflammation and oxidative stress that can lead to the development of obesity-related disorders including CKD [9]. In particular, pro-inflammatory adipocytokines can damage the kidney through alterations in renal hemodynamics resulting in glomerular hyperfiltration, proteinuria and, finally, impairment in GFR [9]. Furthermore, hypertension and type 2 diabetes mellitus (T2DM), two conditions generally associated with obesity, are initiators of renal damage, inducing glomerular hyperfiltration and also cellular damage [10]. Finally, it has been demonstrated that weight loss in subjects with obesity and kidney failure produces a significant reduction in proteinuria and an improvement in GFR [11]. Unfortunately, there is no clinical practice guideline for the management of patients with obesity-related kidney disease.

Diet-induced weight loss, renin-angiotensin blockers, and sodium glucose cotransplorter 2 (SGLT2) inhibitors are currently the main therapeutic measures in subjects with obesity and CKD [12]. In addition, bariatric surgery has been associated with kidney-related beneficial effects in patient with severe obesity [13].

In the last years, different drugs and nutritional protocols have been approved for the treatment of obesity. Among the drugs approved for treatment of obesity, liraglutide at the dose of 1.8 mg has been shown to slow the progression of CKD in subjects with T2DM [14] while a once-daily dose of 3.0 mg is currently indicated for weight loss in people with obesity [15]. Nevertheless, scientific knowledge is lacking about the efficacy and safety of 3.0 mg of liraglutide in patients with obesity and CKD. Of note, tirzepatide is a novel dual gastric inhibitory polypeptide (GIP) and glucagon-like peptide-1 receptor agonists (GLP-1 RAs) approved in the USA for the treatment of T2DM [16]. Recently, an interesting exploratory analysis of SURPASS-4 showed promising effects of tirzepatide on renal function (albumin excretion rate and eGFR) and on a composite renal endpoint (decline of eGFR ≥ 40% from baseline, end-stage renal disease, death from renal failure or new-onset macroalbuminuria) [16]. From the nutritional point of view, many dietary patterns have been proposed to date, but it was demonstrated that any type of dietary intervention leading to weight loss may improve the renal outcomes. However, in recent years, the very low-calorie ketogenic diet (VLCKD) has been increasingly used as an effective tool for weight loss, but absolute contraindications are kidney failure and moderate-to-severe CKD [17–19].

The aim of this manuscript is to review the current evidence on the role of obesity in the development and progression of CKD. In particular, the first part of this narrative review aims to present the main pathophysiological pathways that may link obesity and kidney injury, while the second part reviews the current evidence on nutritional, pharmacologic, and surgical strategies for the management of subjects with obesity and CKD, providing insights into molecular mechanisms of action and clinical effects on the kidney.

### Evidence from Human Studies

Growing evidence suggested an association between obesity and CKD (Table 1). It is noteworthy to mention that proteinuria could also be observed even in the absence of a significant fall in GFR [6]. Indeed, in the CARDIA (Coronary Artery Risk Development in Young Adults) study, a community-based prospective cohort study, 2354 subjects without CKD were included to evaluate the association between modifiable lifestyle-related factors, including obesity, and the risk of kidney disease [20]. During the 15-year follow-up, 77 subjects (3.3%) developed incident microalbuminuria (UACR ≥ 25 mg/g at two or more times). After multivariable adjustment, obesity was significantly associated with microalbuminuria (odds ratio (OR) 1.9, 95% CI 1.1–3.3) [20]. In a cross-sectional study carried out by Qin et al., 41,085 Chinese subjects with obesity and without CKD were enrolled to investigate the correlation between obesity and UACR [6]. The group of subjects with obesity was divided into subjects with peripheral obesity, central obesity, and both peripheral and central obesity. It was observed that obesity was positively associated with UACR. Indeed, subjects with both central and peripheral obesity had a higher risk of elevated UACR, even after adjustment for multiple factors (OR 1.14, 95% CI 1.07–1.12; *p* < 0.001) [6].

**Table 1:** Studies examining the association between obesity and chronic kidney disease

Reference	Design	Aim	Population	Results
**CARDIA study **[20]	Prospective cohort study	Association between modifiable lifestyle-related factors, including obesity, and kidney disease risk	2354 African American and white subjects without microalbuminuria and eGFR > 60 mL/min/1.73 m^2^	Obesity is associated significantly with microalbuminuria (OR 1.9, 95% CI 1.1–3.3)
**Qin et al. **[6]	Descriptive cross-sectional study	The correlation between obesity and the UACR	41 085 Chinese subjects without CKD	Subjects with both central and peripheral obesity had a higher risk of elevated UACR (OR 1.14; 95% CI 1.07–1.12, *p* < 0.001)
**Fox et al. **[22]	Longitudinal cohort study	Identification of predictors of new-onset CKD	2585 subjects without CKD	BMI increased the odds of developing kidney disease by 23% per SD unit (OR 1.23, 95% CI 1.08–1.41)
**Gelber et al. **[23]	Prospective cohort study	Association between BMI and CKD	11 104 healthy men	Each 1-unit increase in BMI was associated with a 5% (95% CI, 3–7%) increase in risk for CKD
**Foster et al. **[21]	Prospective cohort study	Relation between overweight and obesity and the development of stage 3 CKD	2676 subjects	Obesity had 68% increased odds of developing stage 3 CKD (eGFR < 59 mL/min/1.73 m^2^) (OR 1.68, 95%CI 1.10–2.57; *p* = 0.02)
**Hsu et al. **[24]	Retrospective cohort study	Association between increased BMI and risk for ESRD	320 252 subjects	The risk of ESRD is increased in a stepwise manner in subjects with overweight (OR 1.87, 95%CI 1.64–2.14), in subjects with class 1 (OR 3.57; 95%CI 3.05–4.18), in subjects with class 2 obesity (OR 6.12, 95%CI 4.97–7.54), and in subjects with class 3 obesity (OR 7.07, 95%CI, 5.37–9.31)

*BMI* body mass index, *OR* odds ratio, *SD* standard deviation, *CI* confidence interval, *CKD* chronic kidney disease, *ESRD* end-stage renal disease, *UACR* the urinary albumin-creatinine ratio

Several studies reported an association between obesity and the risk for CKD [21–24]. Fox et al. carried out a longitudinal cohort study including 2585 subjects with no evidence of CKD to identify predictors of the development of new-onset kidney disease [22]. Kidney function was estimated by GFR, which was calculated using the modification of diet in renal disease (MDRD) equation. After a mean follow-up of 18.5 years, 244 participants (9.4%) developed kidney disease (eGFR < 60 mL/min per 1.73 m^2^). After multi-variable adjustment, BMI increased the odds of developing kidney disease by 23% per SD unit (OR 1.23, 95% CI 1.08–1.41) [22]. The link between obesity and the risk for CKD has also been found in the Physicians’ Health Study which included 11 104 healthy men [23]. The eGFR was also here calculated using the MDRD equation. It demonstrated that each 1-unit increase in BMI was associated with a 5% (95% CI 3–7%) increase in the risk of CKD (eGFR < 60 mL/min per 1.73 m^2^). In particular, compared with participants in the lowest BMI quintile (< 22.7 kg/m^2^), those in the highest quintile (> 26.6 kg/m^2^) had an OR of 1.45 (95% CI 1.19–1.76; *p* < 0.001) after adjusting for potential confounders [23]. Also, a prospective cohort study enrolling 2676 subjects demonstrated that subjects with obesity had 68% increased odds of developing stage 3 CKD (eGFR ≤ 59 mL/min/1.73 m^2^) (OR 1.68, 95% CI 1.10–2.57; *p* = 0.02) during 18.5 years of follow-up [21]. Finally, in a retrospective cohort study of 320,252 subjects, Hsu et al. found that the risk of end-stage renal disease (ESRD) increased in a stepwise fashion with higher BMI [24]. ESRD was defined as the need for renal replacement therapy. After multivariable adjustment, the relative risk for ESRD was 1.87 (95% CI, 1.64–2.14) in subjects with overweight (BMI 25.0–2.9 kg/m^2^), 3.57 (95% CI 3.05–4.18) in subjects with class 1 obesity (BMI 30.0–34.9 kg/m^2^), 6.12 (95% CI 4.97–7.54) in subjects with class 2 obesity (BMI 35.0–39.9 kg/m^2^), and 7.07 (95% CI 5.37–9.31) in subjects with class 3 obesity (BMI ≥ 40 kg/m^2^) [24].

However, the impact of BMI as a measure of obesity on CKD has not been confirmed in a more recent study [21]. In fact, the Framingham Heart Study (*n* = 212) aimed to characterize the relation between overweight and obesity and the development of stage 3 CKD, and the authors found that the studied association was no longer significant after adjustment for known cardiovascular disease risk factors [21].

### Direct and Indirect Effects of Obesity on the Kidneys

The chronic renal complication of obesity has been named obesity-related glomerulopathy (ORG) [25••]. ORG is a glomerular disease characterized by glomerulomegaly presenting alone or with focal and segmental glomerulosclerosis (FSGS) [26]. The pathogenesis of ORG can be summarized mainly in three aspects: hemodynamic alterations and activation of the renin–angiotensin–aldosterone system (RAAS), adipose tissue-related factors, and inflammation [27]. The mechanism of ORG progression is also varied and very complex, among which podocyte damage caused by chronic low-grade lipid accumulation, compensatory hyperplasia, fibrosis, oxidative stress, and apoptosis is particularly important [27].

Diagnosis of ORG is based on the exclusion of clinical or histopathological evidence of other renal pathology in subjects with a BMI ≥ 30 kg/m^2^ [25••]. The most common clinical presentation of ORG is albuminuria (> 0.3 g/24 h), with or without a larger decline in kidney function (eGFR < 60 ml/min/1.73m^2^) [28]. In addition, ORG has been referred to as hyperfiltration nephropathy because of the central role of glomerular hyperfiltration in the pathogenesis of CKD in obesity [28]. In fact, obesity can damage the kidneys directly by the production of pro-inflammatory adipocytokines as well as indirectly through its systemic complications of obesity, such as T2DM and hypertension [9]. In particular, visceral obesity is reported to damage the kidney via alterations in renal hemodynamics primarily due to vasodilatation of the afferent arteriole and an increased salt reabsorption in proximal tubules [29]. Indeed, vasodilatation of the afferent arteriole is initially caused by the activation of the RAAS [28]. Of note, adipose tissue induces RAAS activation via the secretion of angiotensinogen, mineralocorticoids, mineralocorticoid-releasing factors, and leptin. Furthermore, leptin also stimulates the renal secretion of renin, inducing sympathetic activation [29, 30]. In addition, adipose tissue also secretes cathepsins, which promote the enzymatic conversion of angiotensin I (Ang I) to angiotensin II (Ang II). Overactive RAAS leads to increased levels of aldosterone and Ang II, which promote vasoconstriction in the efferent arteriole, which in turn gives rise to increased transcapillary pressure difference and glomerular hyperfiltration [30]. Moreover, adipocytokines are also involved in regulating vasoconstriction [31]. For instance, asymmetric dimethyl arginine inhibits nitric oxide production, leading to afferent vasoconstriction. Finally, alterations in renal hemodynamics may also be due to an increase in salt reabsorption in proximal tubules, exacerbating both afferent vasodilation and glomerular hyperfiltration due to tubuloglomerular feedback [31]. In particular, sodium filtration load increases resulting in increased sodium reabsorption in the proximal tubules via sodium-glucose cotransporters 1 and 2 (SGLT1 and SGLT2, respectively) [32]. This increased reabsorption of sodium decreases solute delivery to the macula densa, which in turn signals relaxation of the afferent arteriole to increase glomerular filtration pressure (tubuloglomerular feedback) and restore sodium delivery to macular densa. Furthermore, Ang II stimulates the luminal Na^+^-H^+^ exchange and basolateral Na^+^-K^+^-ATPase to activate the epithelial Na^+^ channel and increase proximal and distal sodium absorption. Finally, Ang II also binds directly to the mineralocorticoid receptors, contributing to the reabsorption of sodium and water [32]. The increase in pressure within the glomerular tuft and the retention of sodium and water lead to an increase in renal plasma flow and GFR [32]. In addition, the increase in GFR is also caused by an increase in renal blood flow [33]. In subjects with obesity, blood flow in the kidney is increased by higher extracellular fluid volume and elevated intra-abdominal pressures which increase venous pressure. Furthermore, reduced parasympathetic tone and increased sympathetic activity increase heart rate and cardiac output, thus increasing renal blood flow [33]. Moreover, the increased renal blood flow also increases glomerular filtration pressure [34].

The increased renal plasma flow causes mechanical stress resulting in the expansion of the glomerular basement membrane and glomerular hypertrophy [29]. It has been shown in vitro that the glomerular capillary wall stress promotes reorganization of the podocyte cytoskeleton, resulting in foot process effacement and relative reduction of the glomerular podocyte coating area on the glomerular surface, which in turn compromises the glomerular filtration barrier and serves as a nidus for the development of the proteinuria [35]. In addition, hyperfiltration causes proteinuria [36].

**Fig. 1:**
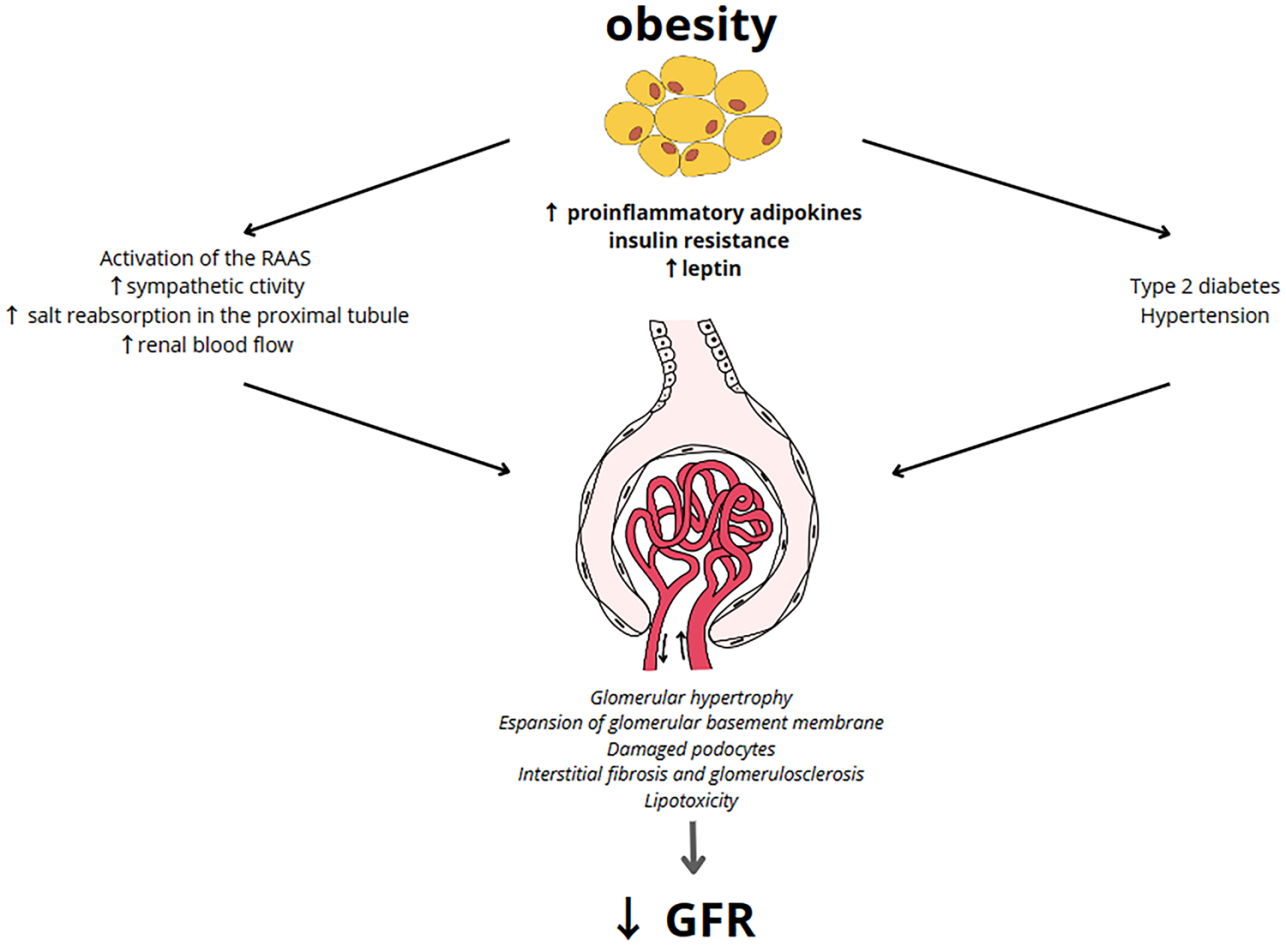
Direct and indirect mechanisms through which obesity may lead to chronic kidney disease. Obesity can damage kidney via the direct effects due to the production of pro-inflammatory adipocytokines leading alterations in renal hemodynamics, and indirectly due to systemic complications of obesity including type 2 diabetes mellitus and hypertension, which are amplifiers of renal damage, increasing glomerular hyperfiltration and inducing cellular damage. In fact, obesity related glomerulopathy has been considered a hyperfiltration nephropathy, resulting in proteinuria and impairment in glomerular filtration rate

The adipocytokines play additional roles in these processes [37, 38]. Leptin exerts a fibrogenic effect by increasing the expression of glomerular transforming growth factor-β1 response and an increase in the production of extracellular matrix components, particularly the production of type IV collagen. This leads to mesangial expansion and glomerular hypertrophy which eventually turns into tubular atrophy, interstitial fibrosis, and glomerulosclerosis [37, 38]. Furthermore, the increase in aldosterone generates reactive oxygen species that may damage podocytes, while decreased adiponectin levels reduce the activation of an energy sensor that is present in podocytes (5′-AMP-activated protein kinase), thereby promoting podocyte effacement and fusion [39, 40]. Finally, excessive production of pro-inflammatory adipocytokines related to obesity causes chronic low-grade systemic inflammation, which is also associated with T2DM and hypertension [41]. Indeed, these conditions can all lead to glomerular hypertension and glomerular hyperfiltration with increased renal plasma flow and impaired autoregulatory capacity, amplifying kidney damage in patients with ORG [42]. In particular, hyperglycemia causes hyperfiltration because it promotes the SGLT2-driven reabsorption of sodium in the proximal tubule and subsequently evokes tubuloglomerular feedback and activation of the RAAS at macula densa in the renal tubule [42]. Systemic hypertension increases glomerular blood flow and promotes irreversible arteriolosclerosis changes that further promote glomerular hypertension and hyperfiltration that can lead to ORG [33]. In addition, hypertension primarily causes glomerular and tubular damage through ischemia [43]. Ischemia can also increase the synthesis and secretion of Ang II, which further constricts blood vessels and leads to the proliferation of renal parenchymal cells [43] (Fig. 1). Moreover, perirenal fat could impair kidney function by increasing interstitial hydrostatic pressure which reduces renal blood flow by direct compression on renal vasculature and parenchyma [44, 45].

### Effects of Body Fat Distribution on the Kidneys

There is evidence that it is not necessarily overweight or obesity per se, but the distribution of body fat, which is correlated with several cardiometabolic disorders and also kidney injury [46, 47]. Indeed, central body fat distribution is correlated with hyperinsulinemia, hypertension, hyperlipidemia, and atherosclerosis [46, 47]. Interestingly, Scaglione et al. showed in a small study that subjects with central fat distribution had reduced renal plasma and blood flow and increased filtration fraction and also albuminuria, while this was not observed in subjects with peripheral fat distribution [48]. However, these were either normotensive or hypertensive subjects, and no comparison was made with lean subjects with central fat distribution [48]. Starting from this point, Pinto-Sietsm et al. studied the relationship between body weight, fat distribution and microalbuminuria, and also elevated or reduced glomerular filtration in 7676 subjects without T2DM [49]. The total population was divided into six groups according to BMI and fat distribution (normal weight, overweight, obesity, and with either central or peripheral fat distribution, respectively). Elevated and diminished filtration were defined as a creatinine clearance ± two times the SD of the creatinine clearance regression line of a group of nondiabetics, peripheral, lean control subjects with UAE of 0 to 15 mg/24 h. Subjects with obesity combined with central fat distribution had a greater risk for microalbuminuria (RR 1.7; 95% CI 1.19–2.35). Subjects with obesity and with either peripheral or central fat distribution had a greater risk for elevated filtration (RR 3.2; 95% CI 1.19–8.47; RR 2.6; 95% CI 1.59–4.28, respectively). Furthermore, subjects with central fat distribution, either normal weight, overweight or with obesity, had a greater risk for diminished filtration (RR 1.9; 95% CI 1.19–3.12; RR 2.0; 95% CI 1.19–3.19; and RR 2.7; 95% CI 1.46–4.85, respectively). Finally, by dividing waist-hip ratio into quartiles, greater waist-hip ratio was associated with a greater risk for diminished filtration, even when corrected for BMI. The authors concluded that a central pattern of fat distribution, not overweight or obesity by itself, seems to be important for renal impairment [49].

Another study of 1555 non-diabetic, middle-aged subjects from the general population aimed to examine the investigated relationship between obesity and two alternative definitions of renal hyperfiltration [50]. Obesity was assessed using the BMI, waist circumference, and waist-hip ratio. GFR was measured by iohexol clearance (mGFR). The dichotomous variables for hyperfiltration were defined as unadjusted (absolute) mGFR (mL/min) above the 90th percentile. The authors used two alternative definitions in which the 90th percentile was specific to age/sex and height or age/sex/height and weight. Only waist-to-hip ratio was consistently associated with hyperfiltration based on both definitions. For the definition based on the age-, sex-, height-, and weight-specific 90th percentile, the association with the waist-to-hip ratio for hyperfiltration was 1.48 (OR 95% CI 1.08–2.02) per 0.10 waist-to-hip ratio increase. The authors concluded that central obesity is associated with hyperfiltration in the general population and that the waist-to-hip ratio may serve as a better indicator of the renal effects of obesity than BMI or waist circumferences [50].

The mechanisms of the adverse renal effect of central obesity are not fully understood, but some effects are known. The central distribution of body fat is characterized by an expansion of visceral adipose tissue (VAT), a key regulator of numerous adipokines and cytokines [51]. VAT is associated with insulin resistance, metabolic syndrome, and T2DM, all pathophysiological processes that are implicated in CKD [52]. Of note, insulin resistance has been associated with structural changes in the kidney, such as mesangial expansion and increased renal fibrosis [53]. Furthermore, VAT may confer hemodynamic effects on the kidney, such as increased filtration fraction and, consequently, glomerular capillary pressure [54]. This evidence suggests that the central distribution of body fat, as opposed to excess body weight distributed more evenly throughout the body, may potentially be more detrimental to kidney function.

### Obesity and the Kidney: Diets

In subjects with obesity, weight loss interventions improve kidney outcomes (i.e., proteinuria and eGFR) [55, 56] (Table 2). Several strategies are available for weight loss and maintenance, such as the modification of lifestyle (diet and physical activity), pharmacotherapy, and surgery, but there is no clinical practice guideline to manage patients with obesity and CKD [31]. The dietary management of subjects with CKD is based on KDIGO guidelines [31]. Many dietary patterns have been proposed to date, but it was demonstrated that any type of dietary intervention leading to weight loss may improve the renal outcomes [56]. Tirosh et al. carried out a dietary intervention randomized controlled trial (DIRECT study) to examine changes in urinary microalbumin and eGFR with various diets, particularly a low-carbohydrate high-protein diet, during 2 years of follow-up [49]. The study included 318 subjects with overweight or obesity, with or without T2DM, and pre-existing mild (*n* = 219; BMI 30.9 ± 3.7 kg/m^2^; eGFR 78.6 ± 15.8 mL/min/1.73 m^2^; UACR 9.7 ± 27.1 mg/g) to moderate renal dysfunction (*n* = 99; BMI 30.9 ± 3.4 kg/m^2^; eGFR 52.6 ± 5.9 mL/min/1.73 m^2^; UACR 18.0 ± 48.7 mg/g). They were randomized to low-fat, Mediterranean, or low-carbohydrate restricted-calorie diets. Significant improvements in eGFR were observed with the low-carbohydrate diet (ΔeGFR + 1.6%; *p* = 0.004), the Mediterranean diet (ΔeGFR + 1.8%; *p* < 0.001), and the low-fat diet (ΔeGFR + 0.4%; *p* = 0.09) with similar magnitude of effect across the diet groups (*p* > 0.05). The UACR also improved similarly across the diets, particularly among participants with baseline microalbuminuria (mean UACR 24.8 ± 51.6 mg/g; *p* < 0.05). In addition, the increase in eGFR was more pronounced in participants with eGFR < 60 mL/min/1.73 m^2^ (+ 7.1%) than in those with eGFR ≥ 60 mL/min/1.73 m^2^ (+ 3.7%) [49]. The Mediterranean diet is characterized by low content of animal proteins and high contents of fibers [57], and it may thus be beneficial in slowing the pace of progression of CKD in subjects with obesity. However, when it is necessary to lose weight, it is important not to overdo the reduction in daily protein intake so as not to encourage excessive loss of lean mass, which is important for maintaining a healthy body composition.

**Table 2:** Effects on the kidney of different approaches in the management of obesity and chronic kidney disease

References	Intervention	Effect
**Diet**		
* Tirosh *et al*. *[49]	Mediterranean diet	↓ albuminuria ↑eGFR
* Bruci *et al*. *[61•], *Barrea *et al*. *[17]	Very low-calorie ketogenic diet	↔ / ↑eGFR
* Tirosh *et al*. *[49]	Low carbohydrate diet	↓ albuminuria ↑eGFR
* Tirosh *et al*. *[49]	Low fat diet	↓ albuminuria ↑eGFR
**Drugs**		
* Marso *et al*. *[65]	Liraglutide	↓ 22% composite kidney outcome * including macroalbuminuria
* Marso *et al*. *[66]	Semaglutide	↓ 36% composite kidney outcome * including macroalbuminuria
* Gerstein *et al*. *[67]	Dulaglutide	↓ 15% composite kidney outcome * including macroalbuminuria
* Heerspink *et al*. *[68••]	Tirzepatide	↓ 42% composite kidney outcome * including macroalbuminuria
-	Naltrexone and bupropion	No evidence
**Surgery**		
* Chang *et al*. *[74]	Bariatric surgery	↓ albuminuria
* Imam *et al. [75]		↑eGFR

*eGFR* estimated glomerular filtration rate, ↓ reduced parameter, ↑ increased parameter,  ↔  stable parameter

*The broader composite kidney outcome including macroalbuminuria consisted of the development of macroalbuminuria, doubling of serum creatinine or 40% or greater decline in eGFR, development of end-stage kidney disease

In recent years, the low-calorie ketogenic diet (LCKD) has been increasingly used as an effective tool for weight loss and the treatment of obesity-related diseases [17, 19, 58, 59]. The VLCKD protocol is characterized by a 600–800 kcal/day with carbohydrate restriction of 30–50 g/day (≃13% of total energy intake), a 30–40 g/day (≃44%) increase in fats, and about 0.8–1.2 g/day proteins/kg body weight (≃43%) [19, 60]. Ketogenic diets are often looked at with concern by clinicians due to the potential kidney harm. In fact, an absolute contraindication of VLCKD is CKD [19]. Although often mistakenly considered a high-protein diet, VLCKD keeps daily protein intake at around 1.2 to 1.5 g/kg of ideal body weight. In addition, VLCKD is based on high biological protein from non-animal and/or animal protein sources, such as peas, eggs, soy, and whey protein. In fact, when supervised by experienced healthcare professionals, the VLCKD might be an option for weight loss in patients with obesity, including those affected by mild kidney failure [17, 19]. A prospective study enrolling 106 subjects with obesity (BMI 34.98 ± 5.43 kg/m^2^) that followed a VLCKD demonstrated that there was no significant change in eGFR from baseline to the end of the ketogenic phase (94.13 ± 19.00 mL/min/1.73 m^2^ vs 89.00 ± 20.83 mL/min/1.73 m^2^; *p* = 0.123) [17]. In addition, Bruci et al. carried out a prospective observational real-life study to evaluate the efficacy and safety of a 3-mo VLCKD on renal outcomes [61•]. Ninety-two subjects with obesity (BMI 33.8 ± 5.8 kg/m^2^; mean eGFR 94.46 ± 18.75 ml/min/1.73m^2^) here included in the study^72^. Based on renal function, the patients were stratified into two groups: subjects with mild chronic kidney disease (MCKD) with an eGFR between 60 and 89 mL/min/1.73m^2^ (*n* = 38; BMI 33.01 ± 6.01 kg/m^2^; eGFR: 76.32 ± 10.4 ml/min/1.73m^2^), and subjects with normal kidney function (NKF), with an eGFR ≥ 90 mL/min/1.73 m^2^ (*n* = 54; BMI 34.46 ± 5.69 kg/m^2^; eGFR: 107.22 ± 11.20 ml/min/1.73m^2^). At the end of VLCKD, no significant change in renal function was observed (from 94.46 ± 18.75 to 95.75 ± 18.52 mL/min/1.73 m^2^; *p* = 0.32). In addition, in the subgroup with NKF, eGFR remained the same (from 107.22 ± 11.20 to 105.28 ± 14.32 mL/min/1.73 m2; *p* = 0.263), while in the subgroup with MCKD, eGFR apparently improved (from 76.32 ± 10.44 to 82.21 ± 15.14 mL/min/1.73 m^2^; *p* = 0.002) [61•].

There is growing evidence that ketones may represent a therapy for kidney disease of various diverse etiology [62]. Ketone bodies, especially β-hydroxybutyrate, in addition to functioning as efficient metabolic fuels, act as signaling molecules influencing several cellular processes [63]. In addition, β-hydroxybutyrate protects the kidneys from acute stress and diseases, as well as aging via suppression of oxidative stress, inflammation, programmed cell death, and fibrosis [62, 63].

Similarly, it has been observed that SGLT2 inhibitors increase the production of ketones (mainly due to the increase in the glucagon/insulin ratio and to increase excretion of glucose), which can be used as a preferred fuel for sodium in the more distal nephrons, necessary to maintain volume status [64]. This process is associated with reduced renal oxygen consumption and, consequently, reduced renal hypoxia and long-term renoprotection [64]. Therefore, these concepts could be potentially translated to ketogenesis induced by VLCKD, and thus, probably this nutritional approach could have a role in the improvement of renal function in subjects with obesity.

### Obesity and the Kidney: Pharmacotherapy

Some drugs approved for the treatment of obesity may have a role in slowing the progression of CKD.

In this regard, given that GLP-1 RAs improve glycemic control and cause weight loss, they are receiving increasing attention for the treatment of both T2DM and obesity. At the same time, evidence of renal efficacy and safety is also emerging.

Results from three cardiovascular outcome trials with GLP-1 RAs, namely “Liraglutide Effect and Action in Diabetes: Evaluation of cardiovascular outcome Results” (LEADER) [65], “Trial to Evaluate cardiovascular and Other Long‐Term Outcomes with Semaglutide in Subjects with Type 2 Diabetes” (SUSTAIN 6) [66], and “Researching cardiovascular Events with a Weekly Incretin in Diabetes” (REWIND) [67], have indicated that patients receiving liraglutide, semaglutide, or dulaglutide, respectively, were at a significantly lower risk of a major adverse cardiovascular event and had a significantly lower occurrence of a composite kidney disease outcome, compared with patients receiving placebo. The risk reductions of the kidney composite outcome observed in LEADER, SUSTAIN 6, and REWIND were 22% (HR 0.78, 95% CI 0.67–0.92), 36% (HR 0.64, 95% CI 0.46–0.88), and 15% (HR 0.85, 95% CI 0.77–0.93) in subjects receiving liraglutide, semaglutide, or dulaglutide, respectively, versus placebo, and these effects were mainly driven by a reduced risk of macroalbuminuria [65–67]. After the exclusion of the macroalbuminuria component, the HR was 0.88 (95% CI 0.68–1.13) with liraglutide and 1.05 (95% CI 0.57–1.93) with semaglutide, compared with placebo [65, 66].

In addition, the REWIND trial with dulaglutide showed a potential effect on eGFR [67]. It was a multicenter, randomized, double-blind, and placebo-controlled trial designed to assess the long-term effect of dulaglutide on renal outcomes. Subjects with T2DM who had either a previous cardiovascular event or cardiovascular risk factors were randomly assigned to either weekly subcutaneous injection of dulaglutide (1.5 mg) (*n* = 4949) or placebo (*n* = 4952) and followed up at least every 6 months for outcomes. The composite renal outcome was defined as the development of macroalbuminuria (development of UACR > 33.9 mg/mmol in people with a lower baseline concentration), a sustained 30% or greater decline in eGFR (i.e., based on two consecutive eGFR concentrations), or new chronic renal replacement therapy comprising dialysis or renal transplantation. Authors reported that the renal outcome developed in 848 (17.1%) participants at an incidence rate of 3.5 per 100 person-years in the dulaglutide group and in 970 (19.6%) participants at an incidence rate of 4.1 per 100 person-years in the placebo group (HR 0.85, 95% CI 0.77–0.93; *p* = 0.0004). The clearest effect was for new macroalbuminuria (HR 0.77, 95% CI 0.68–0.87; *p* < 0.0001), with HRs of 0.89 (0.78–1.01; *p* = 0.066) for a sustained decline in eGFR of 30% or more and 0.75 (0.39–1.44; *p* = 0.39) for chronic renal replacement therapy. These analyses suggested that the use of dulaglutide to reduce glucose concentrations in people with T2DM may confer additional renal benefits. Subsequent studies in a population of subjects with obesity and CKD could open new opportunities for treatment [67].

Finally, we would like to mention tirzepatide, a twincretin recently approved to improve glycemic control in T2DM. Specifically, tirzepatide is an agonist of both GIP and GLP-1 receptors. Interesting findings come from a post-hoc analysis of data from SURPASS-4, a randomized, open-label, parallel-group, and phase 3 study carried out at 187 sites (including private practice, research institutes, and hospitals) in 14 countries [68••]. Eligible participants were adults (age ≥ 18 years), with T2DM treated with any combination of metformin, sulfonylurea, or SGLT2 inhibitor and established cardiovascular disease or a high risk of cardiovascular events. Randomization was 1:1:1:3 to a once-weekly subcutaneous injection of tirzepatide (5 mg, 10 mg, or 15 mg) or a once-daily subcutaneous injection of titrated insulin glargine (100 U/mL) for 104 weeks of treatment. The rates of eGFR decline and the UACR between the combined tirzepatide groups and the insulin glargine group in the modified intention-to-treat population were compared. The kidney composite outcome was time to first occurrence of eGFR decline of at least 40% from baseline, end-stage kidney disease, death owing to kidney failure, or new-onset macroalbuminuria. The mean rate of eGFR decline was − 1.4 mL/min per 1.73 m^2^ per year in the combined tirzepatide groups and − 3.6 mL/min per 1.73 m^2^ per year in the insulin group (between-group difference 2.2 [95% CI 1.6–2.8]). Compared with insulin glargine, the reduction in the annual rate of eGFR decline in the tirzepatide group was more pronounced in participants with eGFR less than 60 mL/min per 1.73 m^2^ than in those with eGFR 60 mL/min per 1.73 m^2^ or higher (between-group difference 3.7 [95% CI 2.4–5.1]). UACR increased from baseline to follow-up with insulin glargine (36.9% [95% CI 26.0–48.7]) but not with tirzepatide (–6.8% [–14.1 to 1.1]; between-group difference − 31.9% [− 37.7 to − 25.7]). A significantly lower occurrence of the composite kidney endpoint was experienced in participants receiving tirzepatide than those receiving insulin glargine (HR 0.58 [95% CI 0.43 - 0.80]) [68••]. In recent clinical studies conducted on people with obesity or overweight with associated conditions, tirzepatide reduced body weight and other cardiorenal risk factors (blood pressure, LDL-cholesterol, glycated hemoglobin, and albuminuria) [69]. Like other kidney-protective drugs, tirzepatide, alone or in combination with SGLT2 inhibitors, caused an early drop in eGFR [70]. In addition, tirzepatide also reduced eGFR slopes in participants with eGFR > 60 ml/min/1.73 m^2^ or with normoalbuminuria [70].

The nephroprotective effects of GLP-1RAs may occur through direct and indirect effects, including improvement of glycemic and blood pressure control. GLP-1RAs attenuate renal fibrosis by suppressing TGF-β and improve glomerular endothelial dysfunction by increasing eNOS phosphorylation and nitric oxide (NO) production [12, 71]. Furthermore, GLP-1 was demonstrated to induce natriuresis and diuresis, inducing phosphorylation and inactivation of Na^+^/H^+^ exchanger 3 (NHE3) in the renal proximal tubules and decreasing circulating concentrations of angiotensin II. Moreover, it has also been shown GLP-1 RA promotes natriuresis by atrial natriuretic peptide secretion from cardiomyocytes in an Epac2-dependent manner [12, 71].

In conclusion, the potential kidney-protective effects of this new drug family should be characterized in detail from the perspective of CKD prevention and treatment.

### Obesity and the Kidney: Bariatric Surgery

In subjects with severe obesity (BMI > 40 kg/m^2^ or BMI ≥ 35 kg/m^2^ associated with obesity-related comorbidity), satisfactory weight loss may not be achievable through intensive lifestyle modification or medications, and therefore, bariatric surgery might be needed [72]. In addition, bariatric surgery can prevent the decline in renal function by reducing proteinuria and albuminuria and improving glomerular hyperfiltration in patients with obesity and impaired renal function [73]. Fathy et al. carried out a prospective study to evaluate the effect of bariatric surgery on albuminuria in 137 patients with severe obesity (mean BMI 54.17 kg/m^2^; mean 24-h urinary proteins 271.61 mg) with no T2DM or hypertension [73]. A statistically significant reduction of the 24-h urinary albumin 6 months after bariatric surgery versus baseline was observed (*p* < 0.001). In addition, albuminuria remission occurred in 83% of subjects [73].

Bariatric surgery may also prevent the kidney function decline in subjects with severe obesity. Chang et al. carried out a study to compare the risk of eGFR decline of ≥ 30% and doubling of serum creatinine or ESRD in 985 subjects who underwent bariatric surgery (BMI 46.4 ± 7.8 kg/m^2^; eGFR 97.2 ± 9.2 mL/min/1.73m^2^) versus controls (*n* = 985; BMI 46.4 ± 8.3 kg/m^2^; eGFR 97.2 ± 2 0.1 mL/min/1.73m^2^) after a follow-up of 4.4 years [74]. The study reported that the subjects who underwent bariatric surgery had a 58% lower risk for ≥ 30% eGFR decline (HR 0.42, 95%CI 0.32–0.55; *p* < 0.001) and 57% lower risk for doubling of serum creatinine or ESRD (HR 0.43, 95%CI 0.26–0.71; *p* = 0.002) as compared to the non-surgery subjects. In addition, the protective effect associated with bariatric surgery persisted in patients with and without baseline eGFR < 90 ml/min/1.73 m^2^, hypertension, and T2DM [74].

There is some evidence on kidney outcomes with regard to the type of bariatric surgery. The observational retrospective cohort study by Imam et al. evaluated changes in eGFR from serum creatinine levels over a median 3-year follow-up period and compared patients who underwent bariatric surgery (*n* = 714; BMI 44.3 ± 6.60 kg/m^2^; eGFR 48.2 ± 10.12 mL/min/1.73m^2^) and non-surgery control patients (*n* = 714; BMI 44.2 ± 7.19 kg/m^2^; eGFR 46.9 ± 10.97 mL/min/1.73 m^2^; serum creatinine 1.5 ± 0.57 mg/dL) [75]. The difference in average eGFRs between patients who underwent surgery and control subjects was significant at 3 months and 2 years (12.58 [95% CI, 10.46–4.70] mL/min/1.73 m^2^ and 12.66 [95% CI, 11.15–14.17] mL/min/1.73m^2^, respectively) and remained significant but somewhat attenuated at 3 years (9.84 [95% CI, 8.05–11.62] mL/min/1.73 m^2^). The Roux-en-Y gastric bypass (RYGB) was associated with a greater effect on renal function compared to sleeve gastrectomy [75]. The weight loss through bariatric surgery reduces the systemic inflammation and peripheral insulin resistance improving blood pressure, dyslipidemia, and T2DM [76]. Apparently, GLP-1 plays a role in improving endothelial function after RYGB and has an antihypertensive natriuretic effect [77, 78].

### Obesity and Kidney: Kidney Transplantation

The prevalence of obesity has tremendously increased over the last decades and the pace of increase in the number of patients with obesity seeking access to kidney transplantation (KT) has followed the same trend [79]. Nevertheless, this scenario has brought several challenges to the management of patients with obesity. The first challenge was to determine whether there is a BMI cutoff to accurately discriminate patients who would obtain survival benefits from KT. Traditionally, many centers have been reluctant to proceed with KT due to several concerns. These included increased risk of perioperative complications in patients with obesity such as deep venous thrombosis, increased risk of re-intubation, poor wound healing, wound infections, hernias, and long-term hospitalization which collectively render a major economic and health burden for a patient with obesity [80, 81]. Given these drawbacks, many centers have chosen not to transplant patients with obesity or considered a BMI of 35 kg/m^2^ as a relative contraindication for KT in the past [82–84]. Therefore, increased BMI served as a gatekeeper for transplant listing for a long time. Over the past two decades, surgical innovation has given rise to the development of minimally invasive robotic technology which gained slowly but steadily popularity worldwide. Robotic-assisted kidney transplantation allowed a drastic decrease in perioperative complications in recipients with obesity a similar graft and patient survival rates when compared with normal weight subjects [85].

Prior observational studies have raised concerns about the risk of delayed graft function (DGF) recipients with obesity [86–88]. A prolonged surgical time that is often needed in recipients with obesity, increased risk of allograft ischemic damage due to obesity-induced sympathetic activation, and hemodynamic instability that is more likely to occur in patients with obesity have been postulated as explanations for the association between obesity and DGF risk [89]. Intriguingly, another meta-analysis (*n* = 21 studies) has noted that the increased risk of DGF ascribed to obesity has relatively decreased in KTs performed after the 2000s [88]. With the advent of novel surgical techniques, concern about operation time and issues related to revascularization may be diminished. Prospective studies looking specifically at DGF, and obesity are needed.

Other controversial issues on the KT of recipients with obesity are graft and patient survival. Some studies suggested that recipients with obesity had a worse graft and patient survival in comparison to normal-weight recipients while others indicated a similar outcome [90–92]. Regardless of the conflicting findings for graft and recipient with obesity survival following KT, the clear survival advantage of KT on dialysis has been firmly established at any level of BMI [93, 94]. Furthermore, the grim prognosis of KT in recipients with obesity tends to subside throughout the years [95]. To this end, the reports from the “older” KT era should be distinguished from the “modern” KT era and be cautiously judged.

Considering emerging evidence, many centers now advocate for the assessment of KT eligibility on an individualized basis considering the frailty, metabolic fitness, functional capacity, and surgical feasibility rather than arbitrary BMI cutoffs. The survival benefit of KT on recipients with obesity is becoming increasingly acknowledged than before. Robust evidence from prospective studies will provide more data on improvements in outcomes and long-term safety.

## Conclusion

Obesity is an independent risk factor for the development and progression of CKD. Unfortunately, there is currently no clinical practice guideline for the management of subjects with obesity and CKD. Weight loss achieved through nutritional, pharmacologic, and surgical approaches can slow the progression of kidney damage and can lead to a significant reduction in proteinuria and improvement in GFR. Further clinical studies are needed to evaluate the efficacy and safety of the kidney regarding the use of liraglutide at a dose of 3 mg per day, the use of tirzepatide, and also VLCKD in the management of subjects with obesity and CKD.
